# Effect of Extracellular Matrix Membrane on Bone Formation in a Rabbit Tibial Defect Model

**DOI:** 10.1155/2016/6715295

**Published:** 2016-03-07

**Authors:** Jin Wook Hwang, Sungtae Kim, Se Won Kim, Jong Ho Lee

**Affiliations:** ^1^Department of New Materials, Oscotec Inc., Seongnam-si 13488, Republic of Korea; ^2^Department of Periodontology, Dental Research Institute, School of Dentistry, Seoul National University, Seoul 03080, Republic of Korea; ^3^Department of Oral and Maxillofacial Surgery, Dental Research Institute, School of Dentistry, Seoul National University, Seoul 03080, Republic of Korea

## Abstract

Absorbable extracellular matrix (ECM) membrane has recently been used as a barrier membrane (BM) in guided tissue regeneration (GTR) and guided bone regeneration (GBR). Absorbable BMs are mostly based on collagen, which is more biocompatible than synthetic materials. However, implanted absorbable BMs can be rapidly degraded by enzymes* in vivo*. In a previous study, to delay degradation time, collagen fibers were treated with cross-linking agents. These compounds prevented the enzymatic degradation of BMs. However, cross-linked BMs can exhibit delayed tissue integration. In addition, the remaining cross-linker could induce inflammation. Here, we attempted to overcome these problems using a natural ECM membrane. The membrane consisted of freshly harvested porcine pericardium that was stripped from cells and immunoreagents by a cleaning process. Acellular porcine pericardium (APP) showed a bilayer structure with a smooth upper surface and a significantly coarser bottom layer. APP is an ECM with a thin layer (0.18–0.35 mm) but with excellent mechanical properties. Tensile strength of APP was 14.15 ± 2.24 MPa. In* in vivo* experiments, APP was transplanted into rabbit tibia. The biocompatible material was retained for up to 3 months without the need for cross-linking. Therefore, we conclude that APP could support osteogenesis as a BM for up to 3 months.

## 1. Introduction

Guided tissue regeneration (GTR) and guided bone regeneration (GBR) are surgical procedures for the treatment of localized periodontal or osseous defects in maxillary or mandibular bone. In order to achieve a successful treatment outcome with GTR or GBR, a barrier membrane (BM), which can prevent unwanted apical migration of epithelial tissue, is required [1, 2].

BMs can be classified into nonabsorbable and absorbable membranes, in clinical studies. Nonabsorbable materials include polytetrafluoroethylene (PTFE), expanded PTFE (ePTFE), and titanium, which are excellent at maintaining space in the oral cavity for bone formation [1, 2]. However, without proper flap closures, they are often contaminated and removed by additional surgery before the ideal healing time. In contrast, absorbable membranes use animal-derived materials or synthetic polymers and do not require surgery for membrane removal, as they are gradually hydrolyzed or enzymatically degraded [3]. Most commercial absorbable BMs use either type I or type III collagen derived from cows or pigs as the composite.

Collagen is accepted as a safe material and also has a nontoxic degradation product [4–6]. However, its degradation period is uncontrolled, with a minimum period of 2 weeks and maximum of 8 months after implantation. Rapid degradation could interrupt bone formation by soft tissue invasion. Thus, to delay degradation, commercial collagen BMs are treated with cross-linking agents, such as 1-ethyl-3-(3-dimethylaminopropyl)carbodiimide (EDC), glutaraldehyde, and formalin [7]. If these remain in the membrane after the cross-linking step, they can induce toxicity and calcification. Moreover, cross-linked membranes can delay revascularization. Therefore, the development of a natural polymer absorbable membrane that does not require a cross-linking agent is urgently needed.

An ideal absorbable membrane should be biocompatible, act as a cell barrier, have mechanical strength, maintain space, integrate with the tissue, have an appropriate degradation time, be convenient to use, and be affordable. The traditional method of membrane production involves the extraction of collagen and reconstruction by cross-linking the agent with the mold. This method, however, has the possibility of destroying native tissue construction and requires the cross-linking agent. Therefore, this study selected an acellular method based on porcine pericardium. This concept retains the tissue structure and is useful for tissue regeneration [8–11].

Porcine pericardium is adaptable to acellular processes. Mild chemicals can be used to prepare an acellular matrix, a process that demands a thin material with low cell density. Raw porcine pericardium is thin (<0.3 mm) and has a low cell density, rendering it ideal for acellular processes. Porcine pericardium's primary degradation product is collagen type I containing 10% hydroxyproline residues. The structure of lyophilized acellular porcine pericardium (APP) is particularly useful for GBR because it has a bilayer structure. The upper layer is very thin (<0.01 mm) and has high density, and it can act as a barrier to tissue invasion. The bottom layer (>0.2 mm) has a microporous structure and can provide spaces for osteoblast homing [12–14]. We hypothesized that chemically treated APP, without cross-linking reagents, could be applied in GBR to prevent soft tissue invasion for over 3 months. We also hypothesized that, after degradation, APP would integrate with the tissue without initiating an immune response in a rabbit tibial defect model.

## 2. Materials and Methods

### 2.1. Fabrication of ECM Membrane

Porcine pericardium (PP) samples were harvested from a slaughterhouse. PP was subsequently cleaned of extraneous tissue and used fresh or stored at −20°C. Raw PP weight was 6 g (15 × 20 cm) and lyophilized weight was 9 g. PP samples were decellularized using a process involving sodium hydroxide (NaOH) and hydrochloric acid (HCl) for about 72 h. PP samples were placed in a solution of 1 M NaOH agitated on a shaker platform, followed by a solution of HCl 0.3%, which was also agitated for about 168 h. Finally, the specimens were rinsed under distilled water (DW) for about 8 h. All steps were performed at 4°C. After being removed from the solution, PP samples were transferred onto wire mesh racks, lyophilized, and freeze dried at 6 mTorr. We got 80 units (3 × 1 cm) after one process.

### 2.2. Sterilization with Ethylene Oxide

After preparation, the collagen membranes were packed. The units were sterilized by exposure to a 100% ethylene oxide atmosphere at a relative humidity of 70% for 8 h at 5°C. After sterilization, the samples were treated with warm (40°C) air flow at atmospheric pressure for 120 h to remove residual ethylene oxide, stored in indicator bags, and sealed.

### 2.3. SDS Page

10 mg of each porcine pericardium and APP were weighed out. The samples were washed thrice with cold normal saline and homogenized in lysis buffer (1 M Tris, 5 M sodium chloride, 0.5 M EDTA, 1% Triton X-100, 1 M DL-dithiothreitol, and 0.1 M PMSF). The homogenate was centrifuged at 13000 rpm for 2 min and supernatant was separated. Protein concentration was determined using a Bradford assay. The samples were run on 6% polyacrylamide gel, and the electrophoresis was conducted at 120 V for 90 min. After electrophoresis, the gels were stained with Coomassie Brilliant Blue R-250 dye in 50% methanol and 7% acetic acid for 24 h. After staining, it was destained in 50% methanol and 7% acetic acid for 60 min and then observed.

### 2.4. Amino Acid Analysis

The amino acid composition of commercial natural collagen membrane and APP were analyzed by Testing & Development Center for Dental Materials of Kyung Hee University. 1 mg of each commercial natural collagen membrane (Bio-Gide®; Geistlich Pharmaceutical, Wolhusen, Switzerland) and APP were weighed out. The PICO-Tag procedure of high performance liquid chromatography (HPLC) was used to analyze PITC-labeled amino acids [15]. The HPLC column was Waters Pico-Tag Column (3.9 × 300 mm, 4 *μ*m). The HPLC instruments (Waters Corporation, MA, USA) consisted of 510 HPLC pump, gradient controller, and 2487 UV detector. The gradient shown in Table 1 was used in the process of analysing amino acids using HPLC.

### 2.5. Morphology

For evaluation of structure, dry samples of each membrane were cut into small pieces (10 × 5 mm) with scissors, applied to a coal carrier, and vaporized with gold at a plating thickness of 30 nm using a low-voltage sputter coater. Subsequently, the surface morphology of the membranes was evaluated using a scanning electron microscope (LEO SUPRA 55; Carl Zeiss AG, Oberkochen, Germany).

### 2.6. Mechanical Tests

Collagen membranes were prepared in strips (30 mm × 10 mm), and uniaxial tensile stress testing was performed using a material testing machine (Universal testing machine; Mecmesin Co., West Sussex, UK). The procedure was performed by repeated loading and unloading at a crosshead speed of 10 mm/min. All tests were conducted at room temperature.

### 2.7. Rabbit Tibial Defect Model and Treatments

A rabbit model was used for* in vivo* experiments after approval was granted by the animal care and use committee of Kyung Hee University (KHMC-IACUC-2012-026). Four rabbits (aged 8 weeks, mean weight 2.5 kg) were used in the study. All animals were provided with food and water once daily.

One gluteal intramuscular injection of 0.5 mL/kg gentamicin (Samu gentamicin; Samu Median Co., Ltd., Seoul, Republic of Korea) was administered to the rabbits. Four holes were drilled bilaterally in each rabbit tibia. The rabbits were divided into two treatment groups those treated with commercial natural collagen membrane derived from porcine skin (Bio-Gide®; Geistlich Pharmaceutical, Wolhusen, Switzerland) and those treated with APP derived from porcine pericardium (LysoGide®; Oscotec, Seongnam-si, Korea). After the holes were made (6 mm in diameter), a polypropylene tube (inner diameter: 2.9 mm, length: 4 mm) was inserted in the hole and covered with a membrane. Histological analysis was performed 12 weeks after the membrane implant.

### 2.8. Evaluation of Histology

Membrane materials must maintain their barrier function long enough to allow osteoblasts to migrate into the tibial defect. At 12 weeks after operation, the defects were visually inspected and biopsies were performed. Images were examined using a light microscope to objectively evaluate membrane degradation, inflammation to host tissue, and new bone formation at the defect site. The four rabbits were sacrificed at 12 weeks after operation using a CO_2_ gas chamber.

The defect and the surrounding soft tissue samples were biopsied and fixed in 70% ethanol for 24 hours, washed in running water for 24 hours, and gradually dehydrated in a series of ethanol solutions ranging from 70% to 100%. The defect samples were then cut into slices (300 *μ*m thickness). The samples were glued to acrylic plates with an acrylate-based cement. The sections were then reduced to a final thickness of ~30 *μ*m by grinding/polishing technique. The sections were stained with hematoxylin and eosin (H&E). The surrounding soft tissue samples were embedded in paraffin and sectioned in 5 *μ*m thick slices followed by hematoxylin and eosin (H&E) staining. The stained specimens were examined under a light microscope (Olympus U-SDO3; Olympus, Tokyo, Japan).

### 2.9. Statistics

All quantitative data were expressed as means ± standard errors of the mean (SEM). All collected data were analyzed using unpaired *t*-tests or one-way analyses of variance (ANOVA) with Tukey's* post hoc* test using Sigma Plot software (Systat Software Inc., San Jose, CA, USA). *P* values below 0.05 were considered statistically significant.

## 3. Results

### 3.1. Sodium Dodecyl Sulfate-Polyacrylamide Gel Electrophoresis (SDS-PAGE)

**Figure 1:**
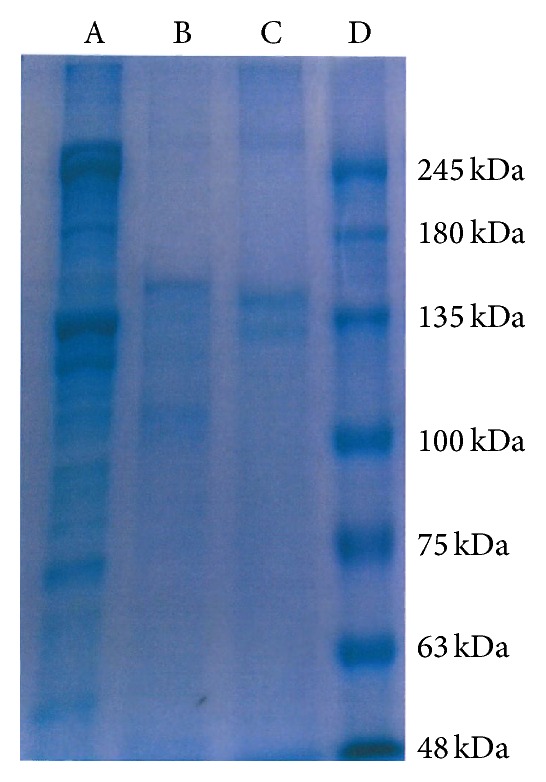
Electrophoretic polypeptide patterns. Collagen type I (A), porcine pericardium (raw) (B), acellular porcine pericardium (C), marker (D).

After treating the pericardium, to prove that the process does not destroy the helical structure of collagen, we performed SDS-PAGE and amino acid analyses. The samples were quantified by Bradford assay (protein content: porcine pericardium 597.68 *μ*g/mL; APP 727.14 *μ*g/mL). An SDS-PAGE gel comparing APP proteins and collagen indicated the presence of bands that match collagens of a lower molecular weight (130 kDa; Figure 1).

### 3.2. Amino Acid Analysis

We conducted amino acid analysis to demonstrate that NaOH treatment does not modify amino acids and lead to the destruction of the intra- and intermolecular collagen bond. The residue patterns of each amino acid in APP were hydroxyproline 11.65%, tyrosine 0.77%, tryptophan 0.19%, and cysteine 0.05%. Thus, APP has similar amino acid ratios to commercial natural collagen membranes (Table 2).

### 3.3. Scanning Electron Microscopy (SEM) Morphological Analysis

**Figure 2:**
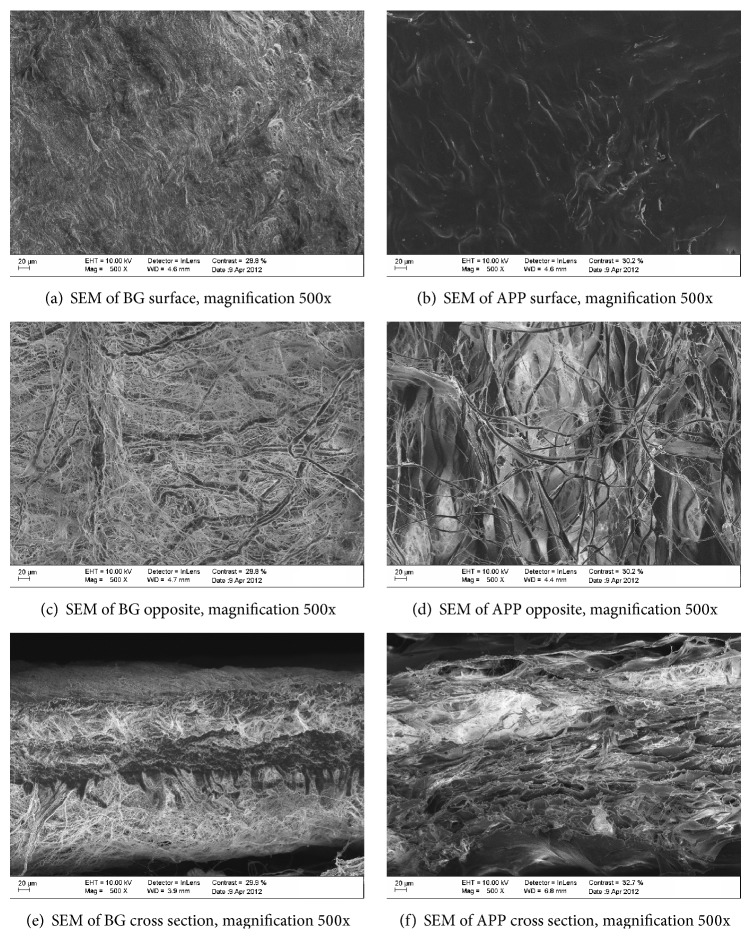
Morphology of Bio-Gide and APP membranes, as visualized by SEM.

In the morphological analysis, distinct differences were noted with respect to the macro- and micromorphology of both groups. Commercial natural collagen membrane showed a bilayer structure with a smooth upper surface and a significantly coarser bottom layer, which was organized mainly in nano- and microsized collagen strands (Figures 2(a) and 2(c)). Further, commercial natural collagen membrane was shown to comprise narrow, individual fibrils (Figures 2(c) and 2(e)). One side of APP showed a high density (Figure 2(b)), whereas the other showed a collagen matrix structure with an interconnected system of pores (Figure 2(d)), which were interlinked via a compact network of three-dimensional collagen strands (Figures 2(d) and 2(f)).

### 3.4. Tensile Strength Testing

**Figure 3:**
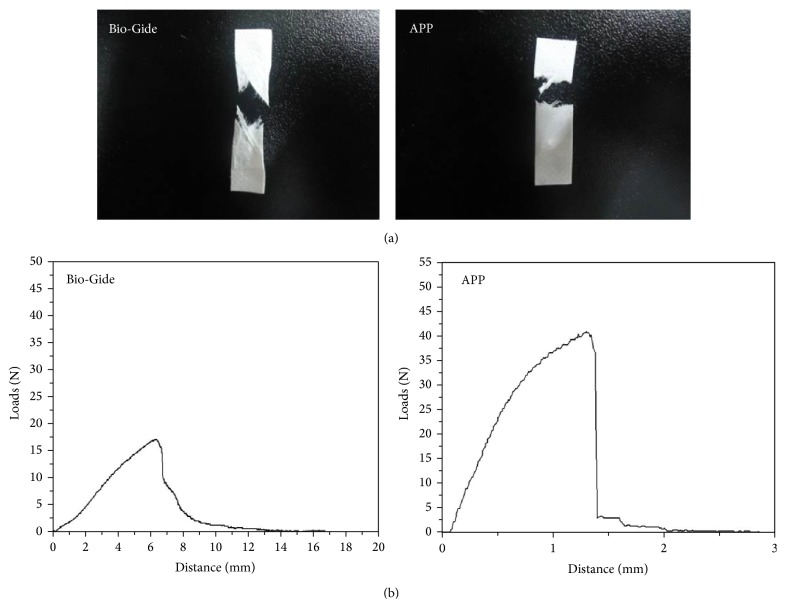
Tensile strength of the membrane (*n* = 10). Optical image of the broken ends (a), graph plotting the results of the tensile strength test (b); see Table 3.

Tests confirmed that APP had a tensile strength similar to that of commercial GBR membranes (Figure 3). The average tensile strength of APP was 14.15 ± 2.24 MPa (see Supplementary Data 4 in Supplementary Materials available online at http://dx.doi.org/10.1155/2016/6715295), whereas that of commercial natural collagen membrane was 6.37 ± 1.35 MPa (Supplementary Data 3, Table 3). The tensile strength of APP was significantly higher than that of commercial natural collagen membrane (*P* < 0.05).

### 3.5. Evaluation of Barrier Function under the Membrane

#### 3.5.1. Immunologic Response to Natural Collagen Membrane and APP

**Figure 4:**
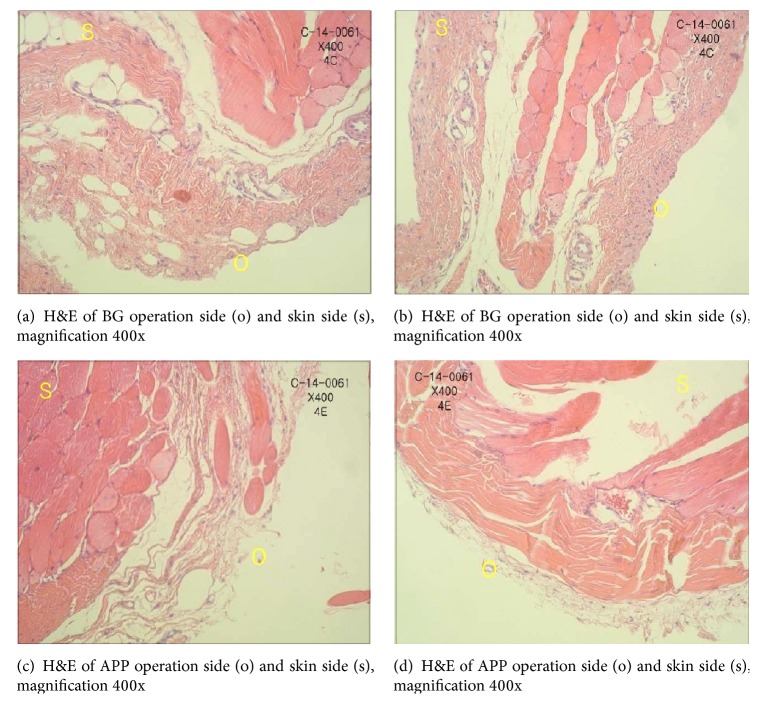
Immunologic response to Bio-Gide and APP.

All animals were clinically healthy, presenting only slight edema in the operation region, which was expected with the surgical procedure performed. Neither group presented any signs of necrosis, suppuration, or infection. At 12 weeks after surgery, the membrane was easy to locate via the previously described polypropylene tube. At 12 weeks after surgery, it was observed that APP was well integrated with the host tissue (Figures 4(c) and 4(d)). Very few macrophages were observed in the host tissue at the operation sites in both groups. In the commercial natural collagen membrane, hematoxylin and eosin (H&E) staining showed no inflammatory cell presence and integrity of the membrane (Figures 4(a) and 4(b)).

#### 3.5.2. Gross Findings

**Figure 5:**
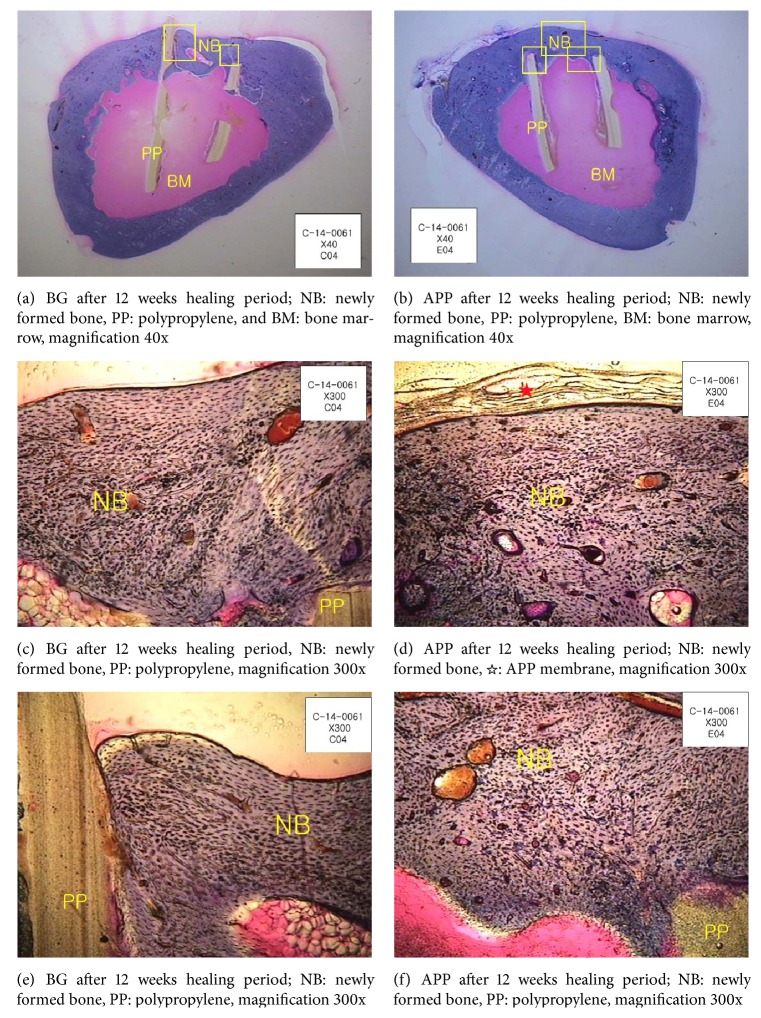
Evaluation of the barrier membrane 12 weeks after surgery.

In general, postoperative healing was uneventful. No signs of postoperative infection or exposure of membrane were observed at the 3-month postoperative follow-up. In the commercial natural collagen membrane group, there was no fibrous tissue invasion under the membrane, and a newly formed bone was typically observed (Figures 5(a), 5(c), and 5(e)). New bone formation was integrated with the old bone areas close to the defect margin in most samples. The APP group also did not exhibit fibrous tissue invasion under the membrane. The gross finding results indicated that integration in the APP group occurred at 12 weeks (Figures 5(b), 5(d), and 5(f)). The commercial natural collagen membranes were mostly degraded at 12 weeks. The membranes in the APP group mostly endured, and morphology showed that they retained their original shape (Figure 5(d)). The APP group also showed the formation of blood vessels and new, mature bone at the operation site.

## 4. Discussion

We investigated whether the application of APP as BM, acellular process-treated porcine pericardium, has a bilayer structure. One side of the bilayer has a high density, whereas the other has a microporous structure (Supplementary Data 2). This structure is thought to be useful for GBR. Moreover, through this experiment, we confirmed that the APP bilayer structure is useful for GBR. The high-density side protects the BM from cell invasion, whereas the microporous side provides space for osteoblast homing. Compared with the commercial natural collagen membrane, APP also has a good tensile strength. High tensile strength is an important function because it allows membrane stabilization with sutures, screws, or tacks. We suggest that APP is provided by acellular process-treated PP structure.

APP has the thinness (<350 *μ*m Supplementary Data 1) and high tensile strength (>5 MPa) of the membrane derived from PP, in addition to being suitable for use as a barrier membrane, as suggested by its previous use as a biomaterial for heart patch and dura mater grafts [16, 17]. Pericardium membranes can be derived from bovine, porcine, and human specimens. Although bovine membranes and allografts carry a risk of transmission of host diseases, porcine tissue has a low risk of transmitting viral disease. We selected PP for the above-mentioned reasons. Our processing of PP rendered it virally inactive.

In order to observe the biocompatibility and biodegradation of APP, we transplanted it in a rabbit tibial defect model. APP remained* in vivo *for over 3 months, while a commercial natural collagen membrane degraded within the same time frame. Microscopic analysis showed an almost complete degradation of commercial natural collagen membrane in three of the four animals at 3 months, whereas APP remained in the area of the operation in three of the four animals (Figure 5(d)). Therefore, we speculate that the APP microfiber is more enzyme resistant than the nanofibers of commercial natural collagen membrane. Twelve weeks after the operation, the process of bone regeneration was active in both the commercial natural collagen membrane and APP groups.

In both groups, we observed new bone formation integrated to old bone areas close to the defect margin in most samples, under the membrane. We tested both groups during bone formation under conditions of cell invasion and bone growth. In addition, in both groups, we observed blood vessel formation and mature new bone at the operation site. Both samples were tested for tissue affinity and bone formation and appeared to be suitable BMs for GBR.

## 5. Conclusion

APP (LysoGide), a biocompatible membrane, lasted for 3 months. Compared with the commercial natural collagen membrane (Bio-Gide), the main advantage of APP was its slow degradation. The shape of the APP membrane remained intact for 3 months in the tibial defect site. On the other hand, the commercial natural collagen membrane was mostly degraded. Although macrophages were observed at the operation site, it is a normal inflammation which is also observed with the commercial natural collagen membrane. We evaluated the rabbits to assess the extent of biodegradation and found that the APP groups demonstrated similar bone healing relative to the control.

In conclusion, it was demonstrated that APP, without a cross-linking agent, can improve bone regeneration and integrate with the natural ECM, revealing its potential as a future biocompatible scaffold for tissue engineering.

## Supplementary Material

Thickness and tensile strength (Supplementary Data).

## Figures and Tables

**Table 1:** Gradient table for amino acids analysis using HPLC.

Time (min)	Flow rate (mL/min)	% A	% B
Initial	1.0	100.0	0.0
9.0	1.0	86.0	14.0
9.2	1.0	80.0	20.0
17.5	1.0	54.0	46.0
17.7	1.0	0.0	100.0
21.0	1.0	100.0	0.0
24.0	1.0	100.0	0.0
25.0	1.0	100.0	0.0

% A: 140 mM sodium acetate (6% acetonitrile).

% B: 60% acetonitrile.

**Table 2:** Amino acid residue patterns in Bio-Gide and APP.

Amino acids (%)	Bio-Gide	APP (LysoGide)
Hydroxyproline	10.26	11.65
Tyrosine	0.77	0.77
Tryptophan	0.22	0.19
Cysteine	0.11	0.05

**Table 3:** Average values of the tensile strength of membranes.

Tensile strength (MPa)	Bio-Gide	APP
	6.37 ± 1.35	14.15 ± 2.34
