# Supplementary material for: Effect of Extracellular Matrix Membrane on Bone Formation in a Rabbit Tibial Defect Model
Source: Biomed Res Int. 2016 Mar 7;2016:6715295. doi: 10.1155/2016/6715295 (PMC4800078; doi:10.1155/2016/6715295)
Supplement: Supplementary file 1 — Thickness and tensile strength (Supplementary Data). [file 6715295.f1.pdf]

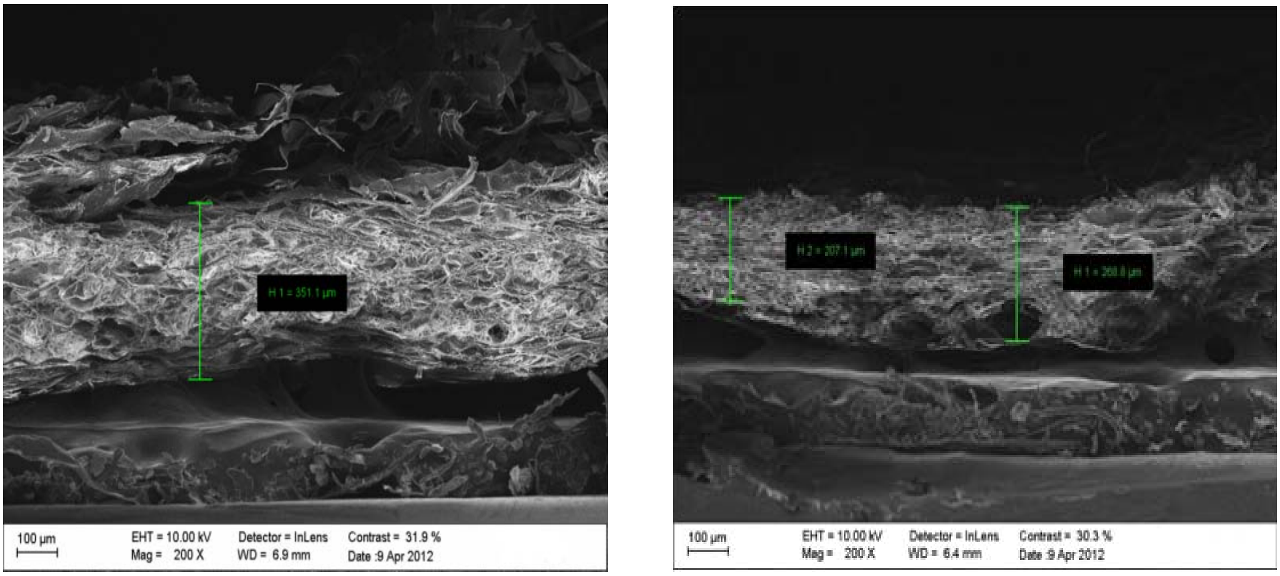

Supplementary Data 1. Thickness of APP, as visualized by SEM.

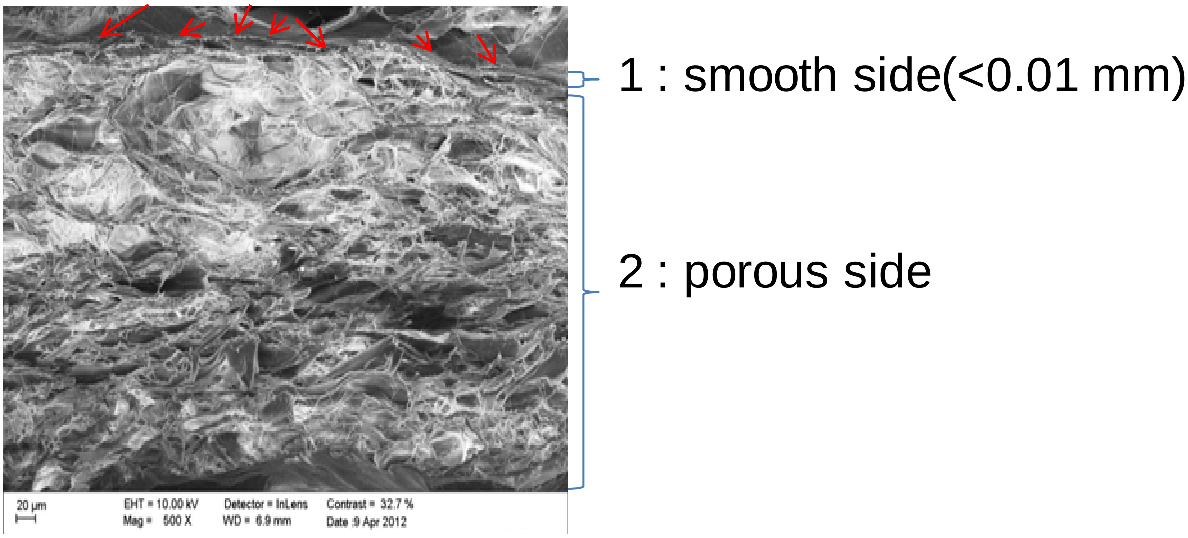

Supplementary Data 2. Morphology of APP, as visualized by SEM.

Supplementary Data 3. Tensile strength of commercial natural ECM membrane.

Test Result		
Results	Tensile strength	6.37 MPa

Tensile Strength Test Result Summary

1. Test Result

Result	Sample 1	Sample 2	Sample 3	Sample 4	Sample 5
1.1. Cross-section area(mm^2)	5.10	4.20	4.50	3.00	4.90
1.2. Maximum load(N)	42.60	27.60	36.20	17.40	26.00
1.3. Maximum deformation amount(mm)	12.48	11.16	13.60	7.86	12.22
1.4. Maximum distortion(N/mm)	3.41	2.47	2.66	2.21	2.13
1.5. Tensile strength(MPa)	3.35	6.57	8.04	5.30	5.31
**Result**	**Sample 6**	**Sample 7**	**Sample 8**	**Sample 9**	**Sample 10**
1.1. Cross-section area(mm^2)	4.00	5.00	2.60	4.10	4.40
1.2. Maximum load(N)	28.40	33.40	18.00	17.20	20.80
1.3. Maximum deformation amount(mm)	11.77	9.62	12.61	12.82	9.86
1.4. Maximum distortion(N/mm)	2.41	3.47	1.43	1.34	2.11
1.5. Tensile strength(MPa)	7.10	6.68	6.92	4.20	4.73
1.6. Average	6.37 MPa (SD : 1.35 MPa, CV : 21.27 %)				

Supplementary Data 4. Tensile strength of APP.

Test Result		
Results	Tensile strength	14.15 MPa

Tensile Strength Test Result Summary

1. Test Result

Result	Sample 1	Sample 2	Sample 3	Sample 4	Sample 5
1.1. Cross-section area(mm^2)	3.40	2.80	2.10	2.10	3.50
1.2. Maximum load(N)	40.60	37.00	36.80	30.80	40.80
1.3. Maximum deformation amount(mm)	1.71	1.55	1.71	1.61	1.25
1.4. Maximum distortion(N/mm)	23.74	23.87	21.52	19.13	32.64
1.5. Tensile strength(MPa)	11.94	13.21	17.52	14.67	11.66
Result	Sample 6	Sample 7	Sample 8	Sample 9	Sample 10
1.1. Cross-section area(mm^2)	3.30	2.70	2.10	2.20	2.50
1.2. Maximum load(N)	48.20	30.20	31.40	31.20	44.00
1.3. Maximum deformation amount(mm)	1.33	1.11	1.14	1.14	1.67
1.4. Maximum distortion(N/mm)	36.24	27.21	27.54	27.37	26.35
1.5. Tensile strength(MPa)	14.61	11.19	14.95	14.18	17.60
1.6. Average	14.15 MPa (SD : 2.23 MPa, CV : 15.32 %)				
